# Prediction of xerostomia in elderly based on clinical characteristics and salivary flow rate with machine learning

**DOI:** 10.1038/s41598-024-54120-x

**Published:** 2024-02-10

**Authors:** Yeon-Hee Lee, Jong Hyun Won, Q.-Schick Auh, Yung-Kyun Noh, Sung-Woo Lee

**Affiliations:** 1 Department of Orofacial Pain and Oral Medicine, Kyung Hee University Dental Hospital, #613 Hoegi-dong, Dongdaemun-gu, Seoul, 02447 Korea; 2 Department of Computer Science, Hanyang University, Seoul, 02455 Korea; 3 School of Computational Sciences, Korea Institute for Advanced Study (KIAS), Seoul, 02455 Korea; 4 Department of Oral Medicine and Oral Diagnosis, Seoul National University School of Dentistry, #101 Daehak-ro, Jongno-gu, Seoul, 03080 Korea

**Keywords:** Medical research, Risk factors, Signs and symptoms

## Abstract

Xerostomia may be accompanied by changes in salivary flow rate and the incidence increases in elderly. We aimed to use machine learning algorithms, to identify significant predictors for the presence of xerostomia. This study is the first to predict xerostomia with salivary flow rate in elderly based on artificial intelligence. In a cross-sectional study, 829 patients with oral discomfort were enrolled, and six features (sex, age, unstimulated and stimulated salivary flow rates (UFR and SFR, respectively), number of systemic diseases, and medication usage) were used in four machine learning algorithms to predict the presence of xerostomia. The incidence of xerostomia increased with age. The SFR was significantly higher than the UFR, and the UFR and SFR were significantly correlated. The UFR, but not SFR, decreased with age significantly. In patients more than 60 years of age, the UFR had a significantly higher predictive accuracy for xerostomia than the SFR. Using machine learning algorithms with tenfold cross-validation, the prediction accuracy increased significantly. In particular, the prediction accuracy of the multilayer perceptron (MLP) algorithm that combined UFR and SFR data was significantly better than either UFR or SFR individually. Moreover, when sex, age, number of systemic diseases, and number of medications were added to the MLP model, the prediction accuracy increased from 56 to 68%.

## Introduction

Dry mouth (xerostomia) and hyposalivation are two separate entities^1^: xerostomia denotes the subjective feeling, the symptom, of dry mouth, whereas hyposalivation is a sign, with a measured decreased saliva flow rate. Xerostomia is associated with decreased salivary gland function. However, only 37% of patients with xerostomia have an actual decrease in salivary flow rate^2^. Ship et al. stated that dry mouth mainly occurs when saliva volume decreases by approximately 50%^3^, but it can also occur within what is considered the normal saliva volume range. While hyposalivation has objective measurement methods for diagnostic judgement, xerostomia is primarily evaluated through screening questionnaires using either single-item approaches or multi-item scales^4,5^.

In older adults, xerostomia is a major concern and significantly reduces quality of life. The exact incidence of xerostomia is unknown, but it ranges from 0.9 to 64.8% in the general population and occurs in approximately 30% of adults over 65 years of age and 40% of adults over 80 years of age^6,7^. The incidence of xerostomia is higher in the geriatric population than in younger adults. According to Fornari et al., the overall prevalence of self-reported xerostomia was 19.1%, but the odds ratio (OR) for xerostomia increased in older patients (≥ 60 years) with chronic diabetes mellitus (OR = 3.59) or those receiving ongoing medication (OR = 2.3)^8^. The causes of xerostomia in the older population have been attributed to local factors, such as poor dental health and infection of the mouth, as well as chronic systemic diseases or autoimmune diseases, including Alzheimer's disease, hypertension, diabetes, Sjögren's syndrome, and rheumatoid arthritis^9–11^. Additionally, the use of medications, adverse effects of drug therapy, and head and neck radiation therapy contribute to the occurrence of xerostomia^12^. In older patients, chronic xerostomia may have multiple oral and dental consequences, such as dental caries, periodontal disease, fungal infections, ill-fitting dentures, and taste alterations^13^. Xerostomia can also seriously impact the quality of life and may alter speech, eating, and swallowing^8^.

Xerostomia is a subjective discomfort in the mouth, and it is difficult to predict the presence of xerostomia based on salivary flow rate alone. Although a consensus has not been reached on the definition of low salivary flow, objective hyposalivation is diagnosed when SFR is below 0.5–0.7 mL/min and UFR is below 0.1–0.2 mL/min^14^. The normal range for SFR is 1.5–2.0 mL/min, while UFR is 0.3–0.4 mL/min^15^. It is commonly assumed that salivary secretion decreases with age; however, the range of salivary flow rates according to age in the older population has not been objectively presented. Furthermore, the SFR in healthy elderly revealed no significant age-related decrease^16^. However, in a recent study, a decreased SFR was associated with cognitive impairment in older patients^17^. With the development of science, medical technology, and medicine, the world's super-aged population is rapidly growing^18^, and previous studies that labeled people over 60–65 years as elderly need to be updated. To date, investigations of salivary flow rate and xerostomia in super-aged seniors over 85 years of age are limited.

The diagnostic field is becoming increasingly smarter. An automatic system and platform based on artificial intelligence that predicts xerostomia in the older population must be developed to reflect the complex clinical characteristics of the older individual. In artificial intelligence (AI) and machine learning, there are two basic approaches: supervised and unsupervised learning^19^. In supervised learning, using labeled inputs and outputs, a model can measure its accuracy and learn over time^20^. In this study, we attempted to predict xerostomia using four supervised learning algorithms including Logistic Regression (LR), Linear Discriminant Analysis (LDA), K-nearest neighbors (KNN), and multilayer perceptron (MLP), increase prediction accuracy, and uncover factors affecting xerostomia and consider xerostomia and non-xerostomia as outputs. Classification is one of the most important areas of machine learning, and LR is the basic method^21^. LDA is also a representative linear classification machine learning technique^22^. The KNN algorithm is a simple, easy-to-implement, supervised machine learning algorithm that can be used to solve both classification and regression problems^23^. MLP is a fully connected class of feedforward artificial neural networks (ANN), that can also present deep features and a network consisting of multiple perceptron layers with threshold activations^24^. Using these four representative supervised learning algorithms, we examined which strategy was the most appropriate for predicting xerostomia in older individuals.

Older individuals experience physiological changes due to aging of the salivary glands and oral mucosa but can also have multiple systemic diseases, be on multiple xerogenic medications, have weakened immunity, and be socially isolated and psychologically depressed^25,26^. Owing to these characteristics, a more complex approach to xerostomia is needed for the older population than in other age groups. This study hypothesized that both oral and systemic conditions may affect xerostomia in older individuals. Furthermore, we wanted to increase the prediction accuracy using an AI machine learning algorithm. By conducting a study on patients aged 1–95 years, we aimed to compare and investigate the predictors of xerostomia in different age groups, including children, adolescents, young adults, and people over 60 years of age.

## Materials and methods

The research protocol for this study complied with the Declaration of Helsinki and was approved by the Institutional Review Board of Kyung Hee University Dental Hospital in Seoul, South Korea (KHD IRB, IRB No-KH-DT21022-002). Informed consent was obtained from all participants. In patients under the age of 18 years, informed consent was obtained from a parent and/or legal guardian.

### Study population

In this cross-sectional study, a total of 829 patients who visited the Department of Oral Medicine and Orofacial Pain at Kyung Hee University Dental Hospital from January 1, 2017, and December 31, 2021 with oral disease and related discomforts were enrolled (71.3% females; mean age, 59.29 ± 16.40 years, 8–95 years). The assessment of xerostomia was conducted through a dichotomous questionnaire to determine its presence or absence^27,28^. The patients were divided into three age groups: 0–29 years (n = 45 patients), 30–59 years (n = 338 patients), and 60–100 years (n = 446 patients). They were also asked to complete questionnaires used for the analysis of sex, age, UFR, SFR, the number of systemic diseases and the number of medications they were taking. Patients who did not agree to proceed with the study, had incomplete data collection, or who dropped out of the study were excluded. Some of the data were previously published using analysis methods that were different from those used in this study^29^.

### Collection of unstimulated and stimulated whole saliva

Prior to the saliva sampling session, the participants were instructed to refrain from caffeine and/or nicotine for at least 4 h and alcohol for at least 24 h. Whole saliva samples, both stimulated and unstimulated, were collected between 9:30 and 11:30 a.m. to minimize diurnal variability; the mean time difference between waking up and collection was 3 h. All participants refrained from drinking alcohol the previous day and were instructed to abstain from eating, drinking, and brushing their teeth before the collection of saliva samples. Unstimulated whole saliva was collected for 10 min using the spitting method. Stimulated whole saliva was collected for the next 5 min while chewing 1 g of gum base, after a 2-min pre-stimulation period to remove saliva retained in the ducts. The UFR and SFR were expressed as mL/min^30,31^.

### The number of systemic diseases and medications

The patients’ general health status and medications were investigated. The presence or absence of systemic disease was dichotomously classified as ‘yes’ or ‘no’. Systemic diseases included high blood pressure, diabetes, hyperlipidemia, digestive disorders, arthralgia, rheumatism, osteoporosis, heart disease, thyroid disease, mental disorders, urinary disorders, and sleep disorders. We also examined the number of medications that the patients were taking for various topical and/or systemic reasons. In the analyses using the number of systemic diseases or medications, the total sum was calculated by replacing 'yes' with '1' and 'no' with '0.’

### Finding cut-off values for xerostomia

First, the dataset was divided into training and test sets with 75% (training set) and 25% (test set) of the total data, ten times (tenfold cross-validation). The assessment of xerostomia was conducted utilizing a dichotomous questionnaire, wherein patients indicated the presence or absence of dry mouth by responding with either 'yes' or 'no' to the following query: "Do you fairly often experience dryness in your mouth?". Subsequently, cutoff values for the dichotomous classification (presence or absence) of xerostomia in each training and test set pair were derived using the Youden’s Index method^32^. Thereafter, the variables were standardized and passed through a logistic regression algorithm. Thus, the cutoff values are the output probabilities provided by the logistic function.

### Receiver operating characteristic curve analysis

In addition to obtaining the cutoff values for xerostomia, the classification performances of the ten different test sets with tenfold cross-validation were measured using the averaged area under the receiver operating characteristic (AUC) curve. A single receiver operating characteristic (ROC) curve was obtained based on the predicted probabilities from the ten sets. This procedure was further applied to sex/age group datasets to investigate differences among them. The age groups were as follows: Group 1 (0–29 years), Group 2 (30–59 years), Group 3 (60–100 years). A post hoc t-test using the AUC curve scores of different age groups and sexes was performed to validate the differences.

### Classifications with multiple features using machine learning algorithms

We performed classification using six features, including the UFR and SFR, which are expected to be related to xerostomia. The four additional features were sex, age, number of systemic diseases, and number of medications. For classification, we used various machine learning algorithms: LR, LDA, KNN, and MLP. A brief description and the experimental settings are as follows: LR and LDA are standard linear algorithms that are widely used in various diagnostic fields^33–35^. The strengths of these algorithms are that they are simple and the learned weights can be used to interpret feature importance. Thus, we used these weights to determine the features that were important for classification. KNN is another simple algorithm that finds the K closest points of a target point and classifies the target as the class that is mainly voted on by the found K neighbor points^36^. The number of neighbors K was optimized to 17. The MLP is a basic neural network with one hidden layer. In addition to the former, this algorithm can capture information from nonlinear structured data. The experimental details for the MLP were as follows: the number of nodes in the hidden layer was 50, and the activation function was a rectified linear unit. To optimize the parameters, the Adam optimizer was used for 1000 epochs with a fixed learning rate of 1e^-3^ and a batch size of 32^37^. All algorithms were tested under tenfold cross-validation, and the fold-averaged AUC scores were used as performance metrics.

### Statistical analyses

Descriptive statistics were reported as mean ± standard deviation or numbers with percentages, where appropriate. To analyze the distribution of discontinuous data, we used the χ2 test, Fisher’s exact test, and the Bonferroni test for equality of proportions. Differences among the three age groups were established based on analysis of variance (ANOVA) for quantitative data. Statistical software programs IBM SPSS Statistics (Version 24.0; IBM, Armonk, NY, USA) and R (Version 4.0.2; R Foundation for Statistical Computing, Vienna, Austria), and the Python package SciPy version 1.4.0. for the machine-learning algorithm was used for all statistical analyses. To show the performance of the classification model at the classification threshold, a ROC curve was plotted, and the AUC value was calculated for each model. As the rule of thumb for interpreting the AUC value, AUC = 0.5 (no discrimination), 0.6 ≥ AUC > 0.5 (poor discrimination), 0.7 ≥ AUC > 0.6 (sufficient discrimination), 0.8 ≥ AUC > 0.7 (acceptable discrimination), 0.9 ≥ AUC > 0.8 (excellent discrimination), and AUC > 0.9 (outstanding discrimination)^38^. Significant predictors of xerostomia were obtained from logistic regression analysis, and the degree to which they contributed (weight) was estimated using weight = log odds ratio values. The correlation between the UFR and SFR was investigated by calculating the correlation coefficient using Spearman's correlation analysis. Statistical significance was set at a two-tailed *p*-value < 0.05.

**Figure 1:**
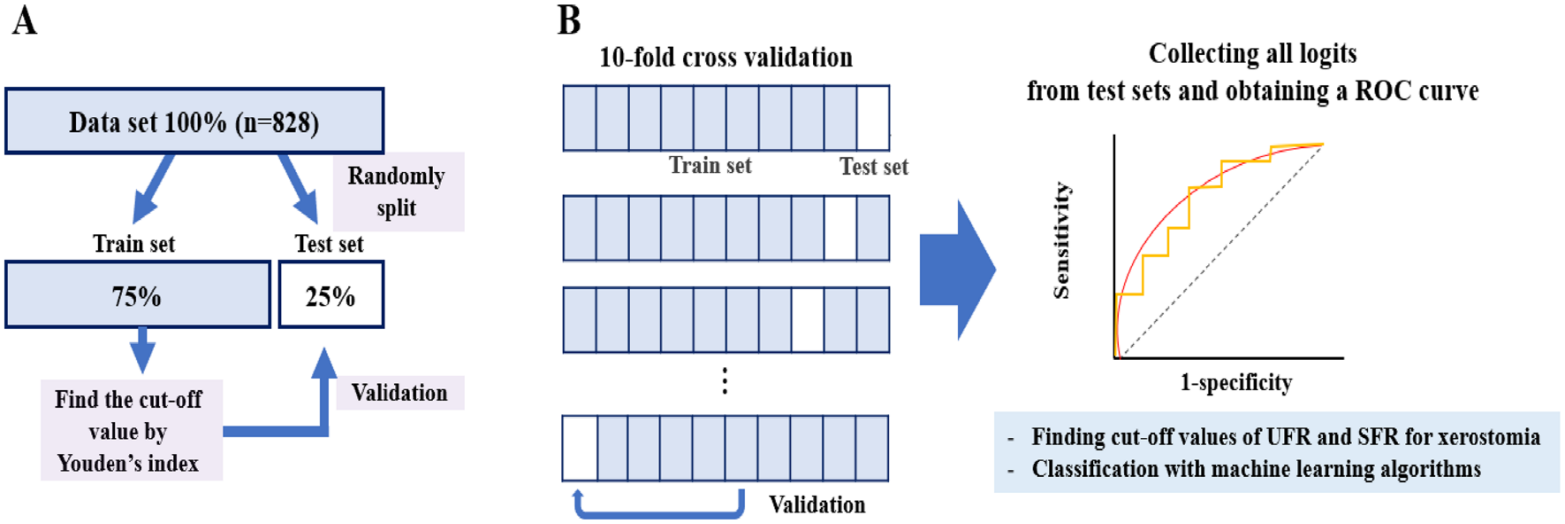
Work flow diagrams of the study. We first find the cut-off values of UFR and SFR by using Youden’s index (**A**). These values are then validated by a randomly split test set. Then, with multiple input variables, classifications using various learning algorithms were performed under tenfold cross validation (**B**). For each algorithm, a ROC curve was obtained from the predicted logits of all ten-fold samples, which represents overall prediction performance of the algorithm.

Significant factors predicting xerostomia were investigated using the four machine learning algorithms and their prediction performances were compared. With this algorithm, we investigated whether there was a difference in the AUC value when only information on the UFR and SFR was provided, and when four additional features (sex, age, number of systemic diseases, and number of drugs) were added. The AUC, sensitivity, and specificity were used to evaluate model performance. After dividing the dataset of 829 patients into a training set and a test set at a ratio of 3:1, machine-learning-based modeling was performed with the training set, and validation of the model was performed with the test set. To increase the prediction accuracy, a tenfold cross-validation was used. With this method, we randomly divided the dataset into 10 parts, with nine being used for training and one for testing. We repeated this procedure 10 times, preserving a different tenth for testing (Fig. 1). This increases the power of the results by increasing the number of samples by 10 times. A *p*-value < 0.05 was considered as statistical significance.

### Ethics approval and patient consent

All participants were given information about the study and provided informed consent. In patients under the age of 18 years, informed consent was obtained from a parent and/or legal guardian. All protocols were approved by the Committee on Ethics of the Kyung Hee Clinical Research Institute, Kyung Hee University Medical Center (IRB no. KH-DT21022002). All methods were performed in accordance with the relevant guidelines and regulations of Kyung Hee University Medical Center.

## Results

### Salivary flow rates and xerostomia according to age groups

**Figure 2:**
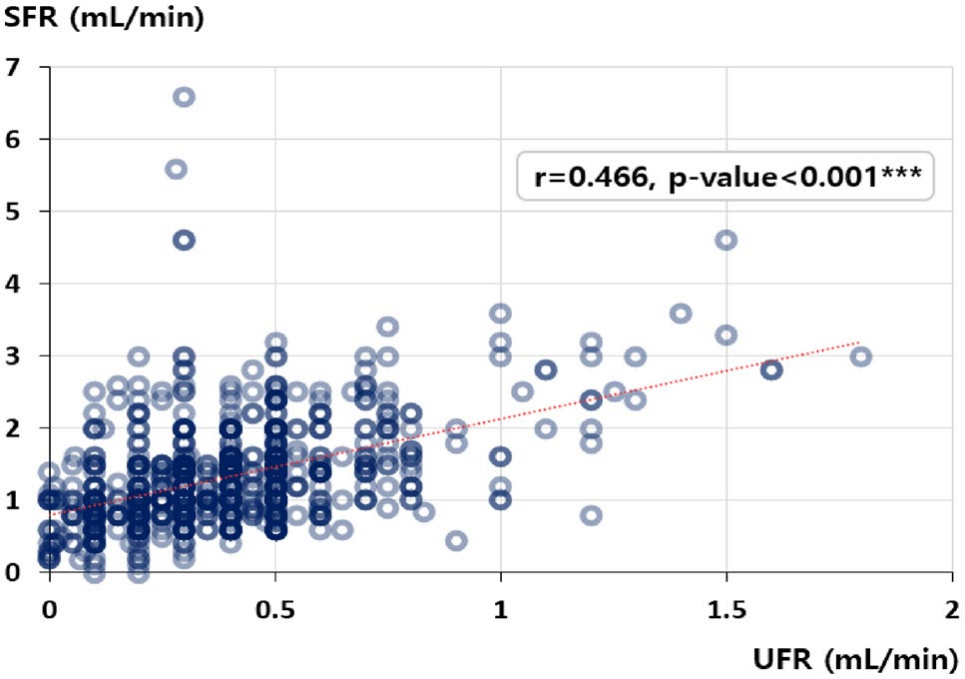
Correlation between unstimulated and stimulated salivary flow rates. The results were obtained using Spearman’s correlation analysis. UFR, unstimulated salivary flow rate; SFR, stimulated salivary flow rate; r, Spearman's correlation coefficient.

A total of 446 patients aged 60–100 years, 338 patients aged 30–59 years, and 45 patients aged 0–29 years completed salivary flow rate measurements and clinical investigations. There was a significant difference in sex distribution by age group: Group 3 (60–100 years) comprised 72.6% women and 27.4% men, whereas Group 1 (0–29 years) comprised 40.0% women and 60.0% men (Table 1). Regarding salivary flow rate, only UFR showed a clear difference depending on age group; UFR decreased with age, and was significantly lower in the following order: Group 3 (0.32 ± 0.20 mL/min) < Group 2 (0.42 ± 0.24 mL/min) < Group 1 (0.64 ± 0.39 mL/min) (*p < *0.001). The UFR and SFR values were moderately correlated (r = 0.466, *p < *0.001) (Fig. 2). The mean SFR was lowest in Group 3 (1.27 mL/min), and Group 1 had the highest value (1.43 mL/min), but there was no significant difference (p > 0.05). In those over 60 years, the mean UFR (0.32 ± 0.20 mL/min) was significantly lower than SFR (1.27 ± 0.86 mL/min) (*p < *0.001). The rate of xerostomia decreased in the following order: Group 3 (29.4%), group 2 (18.6%), and Group 1 (11.1%) (*p < *0.001). In other words, the incidence rate of xerostomia significantly increased with age.

**Table 1:** Demographics, salivary flow rate, and distribution of xerostomia.

Parameter	Group 1 (n = 45)	Group 2 (n = 338)	Group 3 (n = 446)	*p*-value	Post-hoc
	Mean ± SD or n (%)	Mean ± SD or n (%)	Mean ± SD or n (%)		
Age^a^	19.67 ± 6.48	48.79 ± 8.22	71.31 ± 7.58	** < 0.001**	1 < 2, 1 < 3, 2 < 3
Sex^b^					
Female	18 (40.0%)	249 (73.7%)	324 (72.6%)	** < 0.001**	Female: 1 < 2, 1 < 3
Male	27 (60.0%)	89 (26.3%)	122 (27.4%)		
Salivary flow rate					
UFR^a^	0.64 ± 0.39	0.42 ± 0.24	0.32 ± 0.20	** < 0.001**	1 > 3, 2 > 3, 1 > 2
SFR^a^	1.43 ± 0.84	1.39 ± 0.87	1.27 ± 0.86	0.113	
Xerostomia^b^	5 (11.1%)	63 (18.6%)	131 (29.4%)	** < 0.001**	1 < 2, 1 < 3, 2 < 3
Systemic disease					
Number of systemic diseases^a^	0.07 ± 0.33	0.61 ± 0.92	1.34 ± 1.15	** < 0.001**	1 < 2, 1 < 3, 2 < 3
Number of medications^a^	0.22 ± 0.42	0.46 ± 0.85	0.99 ± 1.26	** < 0.001**	1 < 3, 2 < 3

Significant are in value [bold].

^a^ Results were analyzed using ANOVA. ^b^ Results were obtained using χ2 test and repeated χ2 test between two age groups. Group 1 (1): 0–29 years, Group 2 (2): 30–59 years, Group 3 (3): 60–100 years.

### The number of systemic diseases and medications

The average number of systemic diseases for patients was 0.97 ± 1.107 (range: 0–9), and the number of drugs they were taking was 0.73 ± 1.112 (range: 0–6). The number of systemic diseases increased significantly according to age group: Group 1 (0.07 ± 0.33) < Group 2 (0.61 ± 0.92) < Group 3 (1.34 ± 1.15) (*p < *0.001). In other words, as age increased, the number of systemic diseases also increased. There was no statistically significant difference in the number of medications taken between Groups 1 and 2. However, the number of medications (0.99 ± 1.26) in Group 3 was significantly higher than in Group 1 (0.22 ± 0.42) and Group 2 (0.46 ± 0.85).

### The values of UFR and SFR for predicting xerostomia according to age with conventional statistics

**Figure 3:**
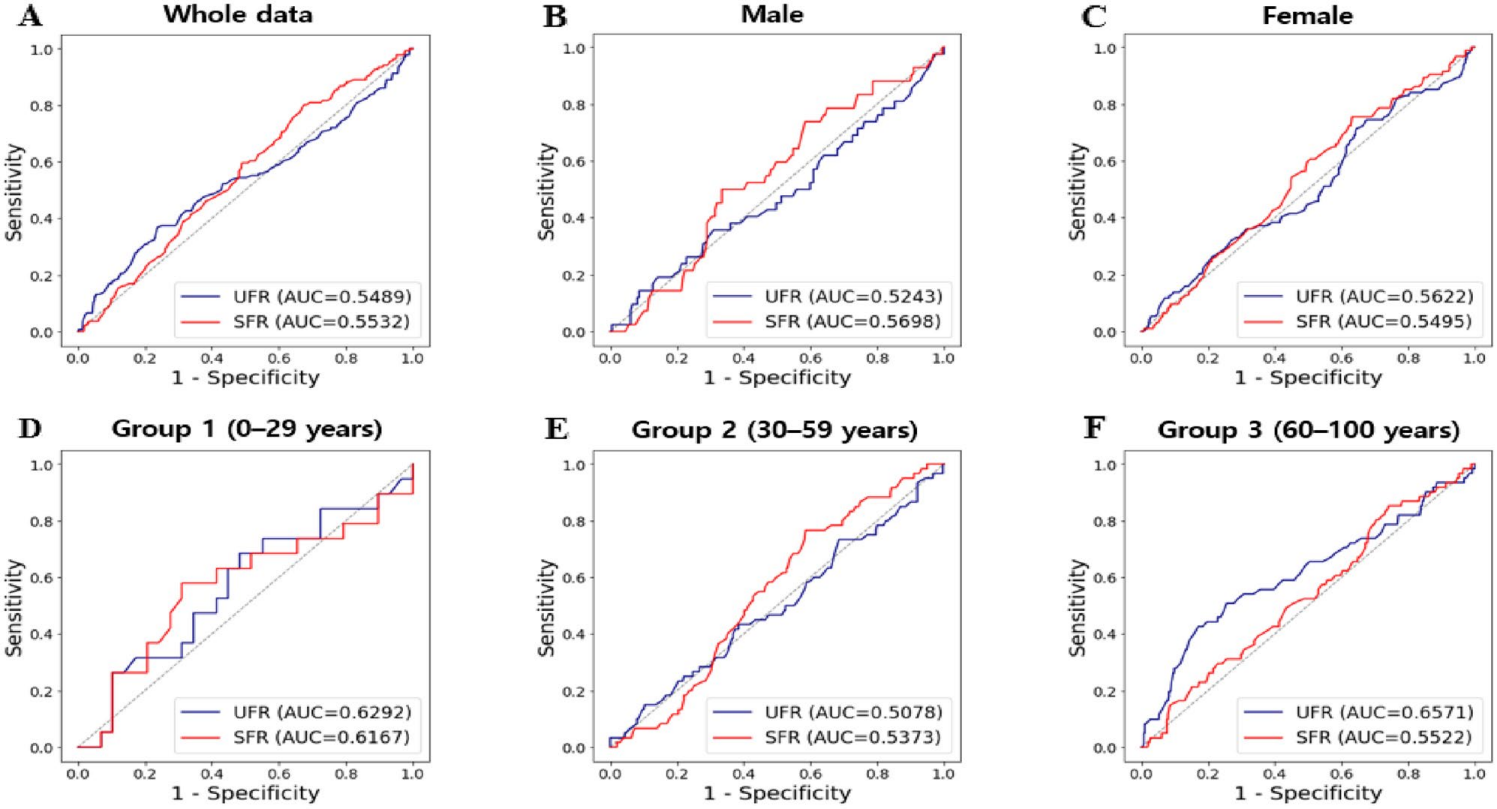
ROC curves of UFR and SFR with conventional statistics. Results of (**A**) all participants, (**B**) male, (**C**) female, and by age group (**D**–**F**). UFR, unstimulated salivary flow rate; SFR, stimulated salivary flow rate; AUC, area under the curve.

To obtain the classification threshold of xerostomia in 829 patients, ROC curves were drawn for the UFR and SFR, and AUC values were obtained (Fig. 3). When using the whole dataset, the AUC value of the UFR (0.5489) was lower than that of the SFR (0.5532). When the data were divided by sex, the AUC values of the UFR were greater than that of the SFR. When comparing the AUC values obtained from the prediction analysis of xerostomia based on the UFR and SFR for the three age groups, the UFR value was greater than that of the SFR in Groups 1 and 3. Among the three age groups, the UFR in Group 3 had the greatest predictive power, and the UFR in Group 3 (AUC = 0.6571) had a significantly higher ability to predict xerostomia than the SFR (AUC = 0.5522) (*p < *0.005).

When the data was grouped by sex or age, the only significant predictive value for xerostomia was the UFR in Group 3 (Table 2), and prediction by SFR was not statistically significant in any group (Table 3). When predicting xerostomia by UFR according to age group, the AUC value of Group 3 was 0.6571 (standard error = 0.0204), the cutoff value of UFR was 0.1409 mL/min (standard error = 0.0013), sensitivity = 74.1%, and specificity = 56.3% (p = 0.0013). In other words, it is meaningful to predict xerostomia using UFR only in individuals over 60 years of age, and it is difficult to predict xerostomia using UFR and SFR data for individuals under 59 years of age.

**Table 2:** AUC and cut-off value for xerostomia with unstimulated salivary flow rate.

Data set		AUC (standard error)	Cut-off (standard error)	Sensitivity	Specificity	*p*-value
All participants (n = 829)		0.5489 (0.0304)	0.1654 (0.0002)	0.7426	0.1272	1.0000
Sex	Male (n = 238)	0.5243 (0.0352)	0.1763 (0.0006)	0.7426	0.1272	1.0000
	Female (n = 591)	0.5622 (0.0421)	0.1604 (0.0003)	0.7128	0.1354	0.5208
Age group	Group 1 (n = 45)	0.6292 (0.0895)	0.3959 (0.0016)	0.2632	0.5172	0.2479
	Group 2 (n = 338)	0.5078 (0.0299)	0.1674 (0.0019)	0.7333	0.2776	1.0000
	Group 3 (n = 446)	**0.6571 (0.0204)**	0.1409 (0.0013)	0.7410	0.5627	**0.0013**

Results were obtained using ROC curve analysis. Group 1: 0–29 years, Group 2: 30–59 years, and Group 3: 60–100 years.

Significant are in value [bold].

**Table 3:** AUC and cut-off value for xerostomia with stimulated salivary flow rate.

Data set		AUC (standard error)	Cut-off (standard error)	Sensitivity	Specificity	*p*-value
All participants (n = 829)		0.5532 (0.0197)	1.0162 (0.0002)	0.2279	0.6460	1.0000
Sex	Male (n = 238)	0.5698 (0.0506)	1.2891 (0.0006)	0.3810	0.5025	1.0000
	Female (n = 591)	0.5495 (0.0253)	1.0117 (0.0005)	0.2660	0.6788	0.7378
Age group	Group 1 (n = 45)	0.6167 (0.0860)	1.0204 (0.0017)	0.3684	0.4483	0.4185
	Group 2 (n = 338)	0.5373 (0.0245)	1.0112 (0.0001)	0.2333	0.6154	1.0000
	Group 3 (n = 446)	0.5522 (0.0355)	0.9824 (0.0004)	0.2951	0.6507	0.7468

Results were obtained using ROC curve analysis. Group 1: 0–29 years, Group 2: 30–59 years, and Group 3: 60–100 years.

Interestingly, the UFR (AUC = 0.6292) and SFR (AUC = 0.6167) for predicting xerostomia in Group 1 (0–29 years) were significantly higher than in Group 2 (30–59 years) (UFR: AUC = 0.5078; SFR: AUC = 0.5373; *p < *0.05). Moreover, the predictive power of SFR for xerostomia was higher in Group 1 than in Group 3 (60–100 years) based on the AUC value (0.6167 vs. 0.5522, *p < *0.01). In summary, the prediction of xerostomia by the UFR was significant in patients aged over 60 years, and the prediction degree of xerostomia by the SFR was the highest in children, adolescents, and young adults, although the difference was not statistically significant.

### Cutoff values for xerostomia by machine learning

**Figure 4:**
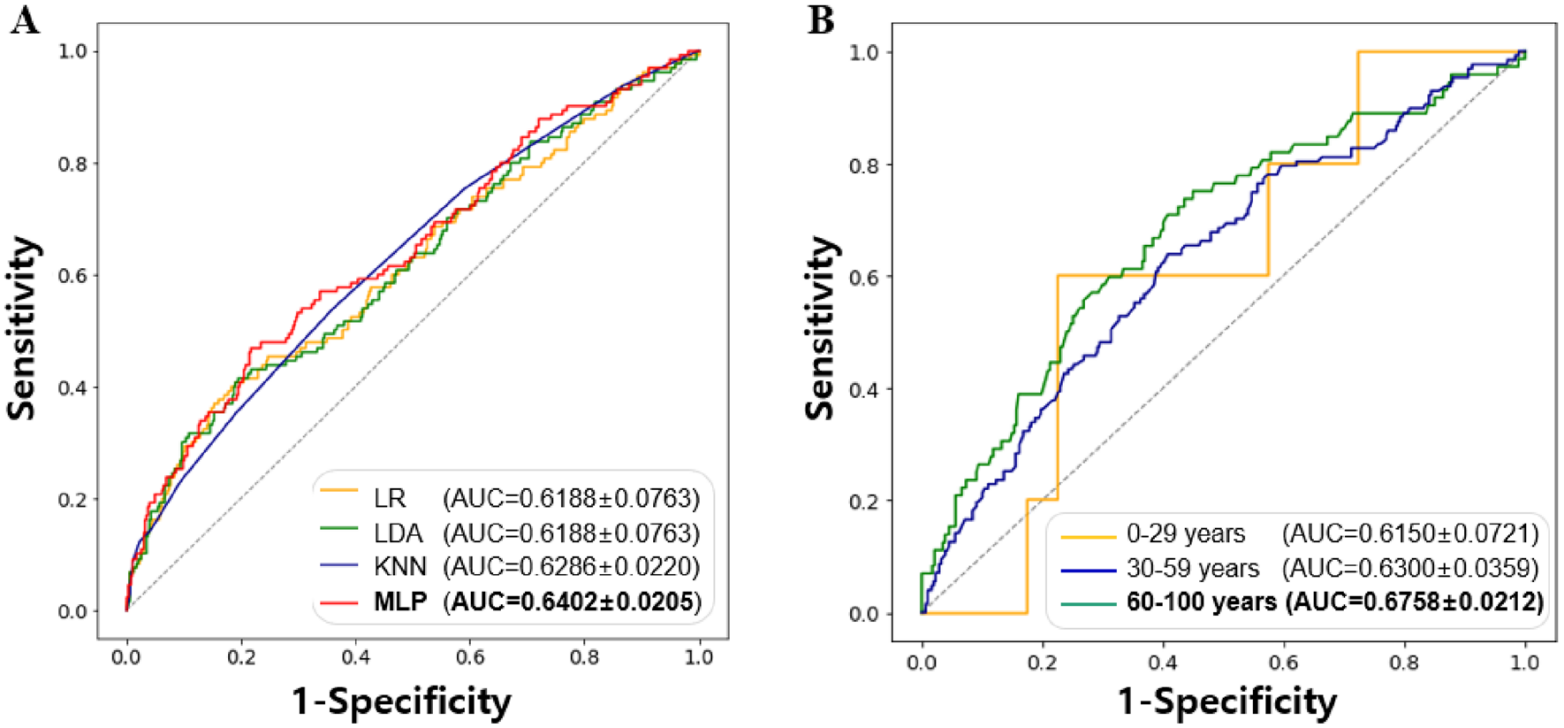
Cutoff values for xerostomia by machine learning models. (**A**) Results using all participants, (**B**) Results divided by age group using MLP**.** LR, Logistic Regression; LDA, Linear Discriminant Analysis; KNN, K-Nearest Neighbors; MLP, Multilayer Perceptron; AUC, area under the curve.

To increase the accuracy of xerostomia prediction based on the salivary flow rate, we used four types of machine learning algorithms: LR, LDA, KNN, and MLP. We attempted to increase the verification power of the results using tenfold cross-validation in each model, which amplified the number of patients in the analysis 10 times (Fig. 4). In the prediction of xerostomia using only UFR and SFR information by four machine learning algorithms, MLP (AUC = 0.6402 ± 0.0205) had significantly higher AUC values than LR (AUC = 0.6188 ± 0.0763) or LDA (AUC = 0.6188 ± 0.0763) (all *p < *0.05), and MLP had higher AUC value than KNN (AUC = 0.6286 ± 0.0220), but the difference was not significant (p = 0.385).

Next, we investigated the predictive power by age group using the MLP algorithm, which had demonstrated the best prediction performance. The results were consistent with conventional statistics, and only in Group 3 (60–100 years) did the URF and SFR-based xerostomia predictions show significance, and the AUC value was the highest (AUC = 0.6758 ± 0.0212) among three age groups. The prediction of xerostomia based on salivary flow rates was not significant in Group 1 (AUC = 0.6150 ± 0.0721) and Group 2 (AUC = 0.6300 ± 0.0359). With the MLP algorithm using UFR and SFR data with tenfold cross-validation, the AUC and prediction accuracy for Group 3 was significantly better than with conventional statistics (*p < *0.05). In other words, combining the UFR and SFR information in the MLP algorithm gave an AUC value (0.6758) significantly better than the AUC values for either salivary flow rate measurement alone (UFR: 0.6571; SFR: 0.5522) (Figs. 3 and 4).

### Main clinical predictors for xerostomia from machine learning

**Figure 5:**
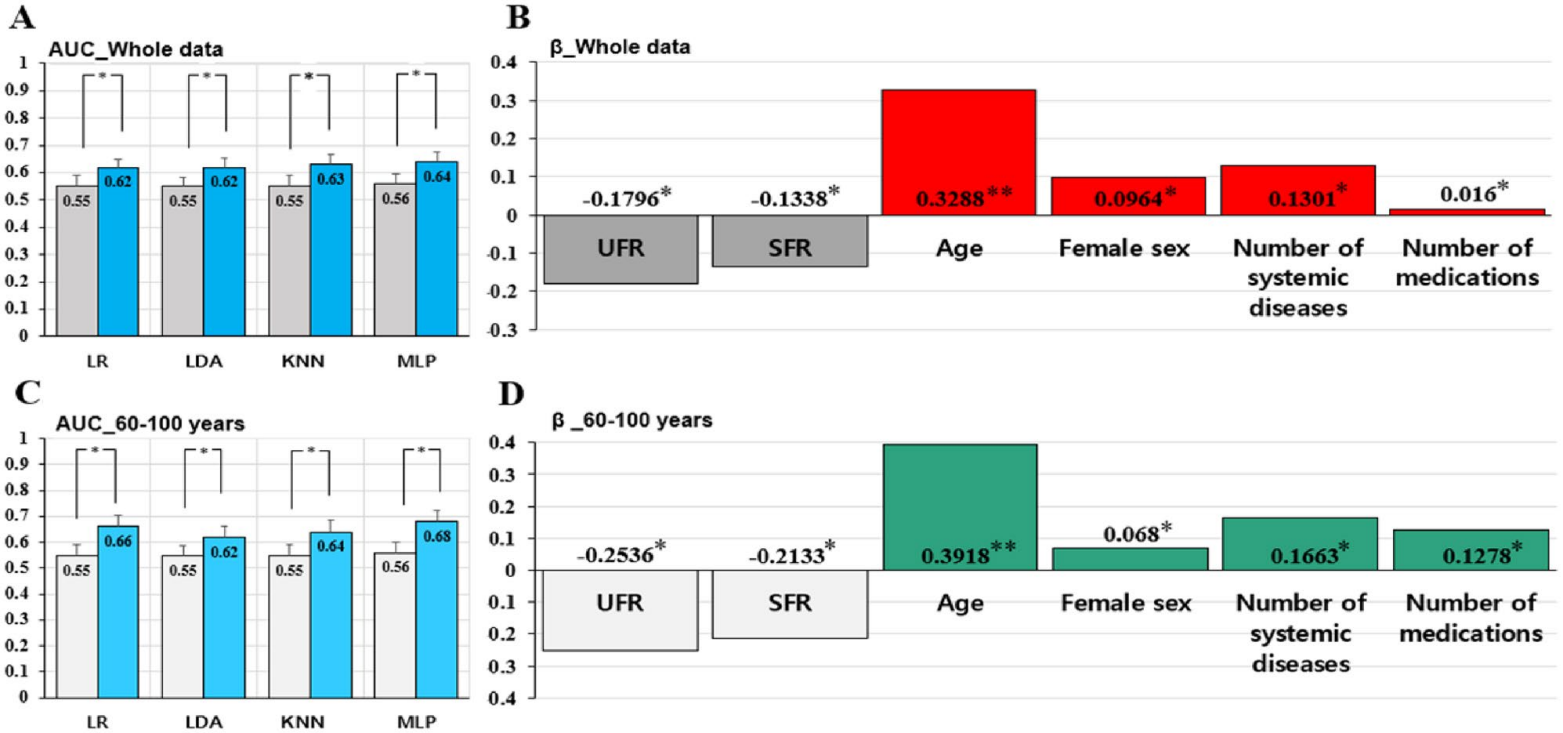
Main clinical predictors for xerostomia when using machine learning algorithms. (**A**, **B**) show analysis of all participants’ data, while (**C**, **D**) show the analysis of Group 3 (60–100 years). (**A**, **C**) The control condition (gray bar) input only two kinds of information: UFR and SFR, while the experimental condition (blue bar) input information on sex, age, number of systemic diseases, and number of medications, in addition to UFR and SFR. AUC values are shown for four machine learning algorithms. Logistic regression analysis (**B**, **D**) indicates the estimated weight (log-odds ratio) values of significant predictors. LR, Logistic Regression; LDA, Linear Discriminant Analysis; KNN, K-Nearest Neighbors; MLP, Multilayer Perceptron; AUC, area under the curve; UFR, unstimulated salivary flow rate; SFR, stimulated salivary flow rate. Statistical significance was set at *p < *0.05, * *p < *0.05, and ** *p < *0.01.

When analyzing all participant’s data, including additional information on sex, age, number of systemic diseases, and number of medications, as well as UFR and SFR, significantly increased the predictive power of the machine learning algorithms for xerostomia. Specifically, in the LR, LDA, KNN, and MLP models, the AUC value significantly increased when xerostomia was predicted the six types of information compared to when xerostomia was predicted using only the UFR and SFR (all *p < *0.05). When the entire dataset was used, the MLP model gave the highest prediction accuracy of 64%. When predicting xerostomia in Group 3, (60–100 years), the machine learning model with the highest prediction accuracy (AUC value) was the MLP model. When only the salivary flow rate (UFR and SFR) was provided to the MLP model, the prediction accuracy was 56%; when all six types of information were provided, the prediction accuracy increased to 68%, and this difference was significant at 13% (*p < *0.05) (Fig. 5).

### Weight of each clinical variable for predicting the xerostomia

Next, we determined the features that were important for the classification by obtaining the learned feature weights from a trained LR algorithm. Figure 5B,D show the feature weights. When using the whole dataset, the main predictor of xerostomia was age (weight = 0.3288, *p < *0.01). The risk of xerostomia increased with age. Female sex was also associated with xerostomia (weight = 0.0964, *p < *0.05). As the number of systemic diseases (weight = 0.1301, *p < *0.05) and medication use (weight = 0.016, *p < *0.05) increased, the incidence of xerostomia increased. Decreases in UFR (weight = -0.1796, *p < *0.05) and SFR (weight = -0.1338, *p < *0.05) were significantly related to an increase in xerostomia (Fig. 5).

When using Group 3 data (ages 60–100 years), a trend similar to that of the whole dataset was observed. In this group, age was weighted the most, followed by UFR, SFR, number of systemic diseases, number of medications, and female sex. That is, the main predictor of xerostomia was age (weight = 0.3918, *p < *0.01), similar to that for the whole dataset. The weight value for the age variable was greater for those aged 60–100 years than when using the whole dataset, indicating that the age variable contributed more to predicting the presence of xerostomia in older people. Female sex was also related to the occurrence of xerostomia (weight = 0.0680, *p < *0.05), and the weight value was smaller than that when using the entire dataset. This suggests that sex has less influence on xerostomia in older individuals. As the number of systemic diseases (weight = 0.1663, *p < *0.05) and medication use (weight = 0.1278, *p < *0.05) increased, the incidence of xerostomia increased. This shows that in older patients, the number of systemic diseases and medications had a greater influence on xerostomia than in other age groups, and that the number of systemic had a greater influence on xerostomia than medications. Decreases in UFR (weight = -0.2536, *p < *0.05) and SFR (weight = -0.2133, *p < *0.05) were significantly related to an increase in xerostomia. It is worth noting that the contribution (weight) of UFR to the prediction of xerostomia was greater than that of SFR in both the whole dataset and for the 60–100 age group. In other words, the UFR contributes more significantly to the prediction of xerostomia than the SFR, and the weight of the UFR is higher in older people.

## Discussion

To our knowledge, no studies have used machine learning to obtain significant predictors of xerostomia in older people and compared their prediction accuracy with that of other age groups. This cross-sectional study was conducted to investigate the prevalence of xerostomia and significant factors associated with these conditions. This study used MLP to investigate how six types of information, including sex, age, number of systemic diseases, and medications, as well as the UFR and SFR, influenced the diagnosis of xerostomia.

Considering the salivary flow rate, only the UFR, but not the SFR, showed a clear decrease with age, and the UFR was a significant predictor of xerostomia in older patients. In people aged 60 to 100 years, the UFR was 0.32 ± 0.20 mL/min, which was significantly lower than that in people aged 30 to 59 years (0.42 ± 0.24 mL/min) and 0 to 29 years (0.64 ± 0.39 mL/min). The normal range of SFR is 1.5–2.0 mL/min and that of UFR is 0.3–0.4 mL/min^6^. In other words, the mean UFR value in individuals aged 60–100 years was within the normal range. The prevalence of xerostomia ranges from 0.9 to 64.8% of the general population^6,7^. There are sex-related differences in the occurrence of xerostomia, which occurs predominantly in women^39^. Sjögren's syndrome, characterized by dry mouth and eyes, and salivary gland fibrosis due to chronic lymphocytic infiltration, is more common in women than in men and may have influenced the female-dominant character of xerostomia^40^. Secondary Sjögren’s syndrome is also associated with autoimmune diseases, such as lupus erythematosus and rheumatoid arthritis, or connective tissue disease^41^, which is also associated with the development of xerostomia. In line with previous studies, the presence rate of xerostomia was significantly higher in Group 3 (60–100 years) (29.4%) than Groups 2 (30–59 years) (18.6%) and 1 (0–29 years) (11.1%). Moreover, when the predictors of xerostomia were investigated using the LR algorithm, female sex was a significant predictor in people aged 60–100 years. Jacob et al. reported that dry mouth commonly occurs in postmenopausal women because of hormonal changes that occur in middle age^39^. Nonetheless, this study emphasizes the importance of closely monitoring dry mouth symptoms not only in middle-aged women but also in older women aged 60–100 years. Interestingly, the strongest predictor of xerostomia was age, both when looking at all participants’ data and when only targeting people aged 60–100 years. Although further investigations are needed to determine which oral and/or systemic conditions govern the development of xerostomia, aging has a detrimental effect on xerostomia development.

UFR and SFR contributed differently to xerostomia prediction. Only the UFR was a significant predictor of xerostomia. The UFR is measured while the patient is at rest, whereas the SFR is measured by spitting out whole saliva while chewing gum to promote saliva production or discharge from the salivary glands to the oral cavity^31,42^. In patients with Sjögren syndrome, 58% had xerostomia, defined by a UFR < 0.1 mL/min, and a UFR < 0.1 mL/min was highly specific for xerostomia^43^. Except for when eating and chewing, the salivary glands are mostly in a resting state^15^. Unstimulated whole saliva reflects the basal salivary flow rate. When studying the UFR of healthy volunteers, young age (< 44 years) was a significant predictor of having an above-average UFR^44^. Thus, a decrease in the UFR was more closely related to subjective discomfort than a decrease in the SFR. Although the UFR has been shown to be useful in predicting xerostomia, SFR is an alternative for patients who have difficulty obtaining saliva at rest or who do not secrete saliva without stimulation^45^. It is worth emphasizing that our study showed that predicting xerostomia using only the salivary secretion rate is meaningful only in people over 60 years of age, and only the UFR is useful. In younger age groups, the prediction of xerostomia by the UFR and SFR was not statistically significant. To further support our findings, integrated or hybrid algorithms should be applied to additional data from multiple races and age groups.

Different authors have developed various machine learning classifiers for human diseases in machine learning repositories. Machine learning algorithms continue to be studied for diagnosing various human oral and systemic diseases^46,47^. Importantly, various machine learning algorithms have their strengths and weaknesses in terms of the amount of data required, impact of outliers, computation speed, dimensionality, handling of missing data, overfitting tendency, and explanatory power^20,48^. Therefore, to select the optimal machine learning model for each disease, multiple models are tested simultaneously, and their prediction accuracies are compared. In this study, four types of classifiers were attempted and tenfold cross-validation was performed on all algorithms to prevent overfitting of the data^49^. An MLP, a type of ANN, is a network composed of multiple layers, including an input layer, multiple hidden layers, and an output layer^50^. The MLP yielded the best accuracy in this study. MLPs containing more than two hidden layers are referred to as deep MLPs; the MLP used in this study had seven hidden layers. In clinical practice, the MLP has yielded better classification outcomes than conventional statistics^51^. An MLP has the advantage of distinguishing data that are not linearly separable, compared with the logistic regression model suitable for traditional classification^52^. Additionally, each layer of neurons activates sequential layers, eventually producing output variables in the output layer^53^. Thus, the MLP performance is powerful because it can amplify aspects of the input that are important for classification while suppressing irrelevant information^54^. However, the salivary flow rate features used in this study remained at a prediction accuracy of 62–68% even when based on the most appropriate machine learning algorithm, an MLP. This is a very low prediction accuracy compared to the 90% accuracy obtained when the MLP classifier was used for other diseases^55,56^. It can be concluded that it is difficult to predict xerostomia based on the salivary flow rate in older patients, even with AI-based diagnostics. It is expected that the composition and properties of saliva, condition of the oral mucosa, and other diseases will combine to have a complex effect on the occurrence of xerostomia in the elderly.

In this study, the prediction accuracy of the MLP significantly increased with the number of input features. Generally, adding more data or features to a machine-learning model can increase its accuracy^57^. An MLP has no restrictions on the type or number of input variables^58^. In addition, an MLP increases the complexity and classification capabilities by considering all possible interactions between input variables^59^. In this study, when six features were input, the MLP performance increased by 12% compared with when two features, UFR and SFR, were input. In addition, when predicting xerostomia with LR, age had a higher weight value than salivary flow rate, demonstrating that it was a more important predictor. This suggests that even if the optimal algorithm for disease diagnosis is identified, the simultaneous application of two or more algorithms or a convergence approach may be necessary for a more accurate understanding of the disease^60^. Theoretical research is required to evaluate the ability of various models to handle high interaction orders, the impact of factors on model performance, and the explanatory power of the results.

Studies on the AI-based diagnosis of xerostomia in older patients are extremely limited. Xerostomia is a significant burden in geriatric populations. In particular, dry mouth can affect oral health and everyday movements such as speaking, chewing, and swallowing^61^. In older individuals, xerostomia associated with reduced salivary flow may be related to dental caries, oral mucosal diseases such as oral candidiasis, recurrent aphthous ulcers, changes in taste, halitosis, or burning mouth syndrome^6,62^. Therefore, changes in the salivary flow rate and/or the presence of xerostomia can lead to deterioration in the patient’s quality of life. Conversely, systemic conditions such as hypertension, hyperlipidemia, diabetes mellitus, obesity, radiotherapy of the head and neck, and autoimmune diseases related to decreased function of the exocrine glands can affect salivary glands, saliva secretion, and oral health^63–65^. We found that multiple systemic diseases were the main predictors of xerostomia in older individuals. Polypharmacy is a significant predictor of xerostomia. Many drugs also cause xerostomia, including antihypertensive drugs, nonsteroidal anti-inflammatory drugs, steroids, hypoglycemic drugs, anticoagulants, antidepressants, multivitamins, and supplements^66–68^. Other risk factors for xerostomia in older individuals include malnutrition and psychosocial problems such as depression, anxiety, and stress^69^. Continued efforts are needed to develop machine learning algorithms that can predict xerostomia by comprehensively considering these factors in older individuals.

This study had several limitations. First, as we did not target healthy volunteers, we must be cautious in interpreting the results of this study to those of the general population. This study targeted patients who visited the hospital complaining of mouth discomfort and who agreed to have their salivary secretion rates measured. Second, because this study was conducted at a single institution, additional experiments at multiple institutions are required to ensure the validity and reliability of the results. Third, only six input features were considered when evaluating xerostomia. Each systemic condition and the type of medication (i.e., whether they are xerogenic or non-xerogenic drugs) may have varying impacts on xerostomia. However, this study did not consider individual systemic conditions and types of medication. As the model's prediction accuracy ranging from 56 to 68%, further research should include the patient's detailed oral condition, general condition, psychological condition, and sleep-state information as input features.

## Conclusion

The prevalence of xerostomia in older individuals was higher than that in children, adolescents, and young adults, and age was the most important predictor of xerostomia. In those aged 60–100 years, xerostomia could be significantly predicted by the UFR; however, in other age groups, there was no statistical significance in predicting xerostomia using the UFR alone. Among the machine learning algorithms, the MLP was the most suitable for predicting xerostomia. In this model, the predictive power was higher when additional clinical information, including sex, age, number of systemic diseases, and medications, was provided rather than when only salivary flow rate was provided. Predicting xerostomia in everyone solely based on salivary flow rates may not be feasible. Since forecasting xerostomia using a single indicator or fragmented aspects in patients is challenging, an AI algorithm that takes a comprehensive approach to patients is necessary. Absolutely, given the model's prediction accuracy ranging from 56–68%, it's essential to exercise caution while interpreting the study's findings. To solidify our research results, we need to continue multicenter studies with large samples while considering the composition of input features or participants.

## Data Availability

The datasets used and/or analyzed in the current study are available from the corresponding author upon reasonable request.
